# Continuous cardiac output measured with a Swan‐Ganz catheter reacts too slowly in animal experiments with sudden circulatory failure

**DOI:** 10.1002/ame2.12235

**Published:** 2022-06-06

**Authors:** Sigríður Olga Magnúsdóttir, Carsten Simonsen, Bodil Steen Rasmussen, Peter Enemark Lund, Benedict Kjærgaard

**Affiliations:** ^1^ Biomedical Research Laboratory Aalborg University Hospital Aalborg Denmark; ^2^ Department of Clinical Medicine Aalborg University Aalborg Denmark; ^3^ Department of Cardiothoracic Surgery Aalborg University Hospital Aalborg Denmark; ^4^ Department of Anesthesia and Intensive Care Aalborg University Hospital Aalborg Denmark; ^5^ Unit of Clinical Biostatistics Aalborg University Hospital Aalborg Denmark

**Keywords:** cardiac output, flow probe, hemodynamics, pigs, Swan‐Ganz catheter

## Abstract

**Background:**

In many animal experiments, it is vital to detect sudden changes in cardiac output (CO). This porcine study compared CO that was measured with a Swan‐Ganz pulmonary catheter with the gold standard (which was a transit‐time flow probe around the pulmonary artery) during interventions that caused hemodynamic instability.

**Methods:**

In one series, 7 pigs were exposed to sudden changes in CO. In another series, 9 pigs experienced more prolonged changes in CO. All the pigs had a Swan‐Ganz catheter placed into the pulmonary artery and a flow probe around the pulmonary artery. Adrenaline infusion and controlled hemorrhage were used to increase and decrease CO, respectively. The measurements of CO before and after each intervention were compared for correlation, agreement, and the time delay that it took each method to detect at least a 30% change in CO. A Bland–Altman test was used to identify correlations and agreements between the methods.

**Results:**

In the first series, there was a delay of 5–7 min for the Swan Ganz catheter to register a 30% change in cardiac output, compared with the flow probe. However, during prolonged changes in CO in the second series, there was a good correlation between the 2 methods. Mixed venous oxygen saturation reacted faster to changes than did CO; both were measured via the Swan‐Ganz catheter.

**Conclusions:**

In many animal studies, the use of Swan‐Ganz catheters is suitable; however, in experiments with sudden hemodynamic instability, the flow probe is the most advantageous method for measuring CO.

## Graphical abstract

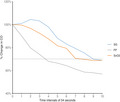

## 1 INTRODUCTION

Cardiac output (CO) is an important hemodynamic variable and is the major determinant of oxygen delivery and blood pressure. In many animal experiments, it is vital to detect sudden changes in CO. There are many methods to monitor CO, which vary in their invasiveness and continuousness, and some of them can also monitor other parameters. Moreover, all these methods may have advantages that are dependent on the experiment that must be performed.

From the time that Swan and Ganz described the use of right heart catheterization with a balloon‐tipped catheter in 1970, the use of a Swan‐Ganz (SG) catheter has become widespread.^1^, ^2^ The principle is based on Fick’s technique that was described 100 years earlier.^3^ This pulmonary artery catheter uses thermodilution and a computer‐calculated estimate for CO. The system that we used performed a measurement every 54 s; on the basis of this method, average CO was calculated. This is a commonly used system in human intensive care units; in contrast to the first system, it is semicontinuous and allows for the monitoring of CO over time, measurement of mixed venous oxygenation, measurement of blood pressure on the right side of the heart, and an estimate of the pressure in the left atrium. However, the system is invasive, with catheters inserted via the veins to the right side of the heart and further inserted to the pulmonary artery. There is some delay in the reactions to sudden changes in the parameters, and the monitoring of left atrial pressure is an indirect evaluation based on an intermittent pulmonary capillary wedge pressure test. In 25‐year‐old experiments in sheep, there was a clinically important time delay in the detection of CO changes in response to sudden interventions that affect the circulation.^4^ As a reference, the researchers used a transit‐time ultrasound blood flow probe (FP) around the pulmonary artery. The transit‐time ultrasound method has been described as being very accurate.^5^

This specific procedure may be the most accurate method, but it is also the most invasive, and it is often referred to as being the gold standard.^6^, ^7^ In other experiments, a flow probe around the ascending aorta was used, but this method cannot measure the circulation in the coronary arteries, which, in many experiments with toxic agents or hypoxia, can vary by up to 300%.^8^, ^9^, ^10^, ^11^

We are aware that there are other methods for continuous CO monitoring, such as pulse wave analysis and the PICCO monitor.^12^, ^13^, ^14^ The intermittent monitoring of CO via transthoracic echocardiography by trained personnel is also possible in humans.^15^ The concentration of carbon dioxide (CO_2_) in end‐tidal expired air is often used as a substitute for cardiac output changes; in a new and more sophisticated method, it seems possible to measure CO via the capnodynamic determination of CO_2_ before and after the blood passes the lungs by constantly changing the relationship between inspiration and expiration. This procedure has been tested in both animals and small children undergoing cardiac surgery.^16^, ^17^, ^18^ Furthermore, it is possible to measure CO by using a method involving a small arteriovenous shunt with stable blood flow and with intermittent saline dilution into the venous side. Ultrasound detectors that measure blood from the artery and the blood returning to the venous side after a saline bolus make it possible to calculate CO. This method appears to be promising, especially in small children and in small animals, wherein a SG catheter cannot be used because of the size.^7^, ^19^, ^20^ Moreover, there is a well‐established standard for accuracy in CO monitoring, with a percentage error of no more than 30%^21^; however, we are not aware of any standard for acceptable delays in the measurements.^22^, ^23^, ^24^ In the present study, errors greater than 30% were considered unacceptable.

In a recent porcine experiment with carbon dioxide poisoning, we used a highly invasive method of measuring CO and pulmonary vascular resistance (PVR), with a transit‐time flow probe being used around the pulmonary artery and direct pressure monitoring being used in the left atrium and in the central aorta after sternotomy.^25^ In this type of study using life‐threatening intoxication, it was important to immediately detect a serious decrease in CO, and we were not convinced of the simultaneous use of SG catheters in that experiment. For this reason, we decided to compare the use of SG catheters with the gold standard with a transit‐time FP around the pulmonary artery in both situations; additionally, we aimed to consider sudden changes in CO and the use of more prolonged measurements. For this study, we used pigs because their size allows the use of much of the equipment used in human clinics and because we have experience with pigs from other experiments.

The null hypothesis of the study was that the use of a SG catheter and the use of a transit‐time flow probe around the pulmonary catheter are comparable in precision, especially in the first minutes after serious events affecting CO; additionally, we hypothesized that would be no large bias.

## 2 METHODS

### 2.1 Animals

All experiments were performed at the Biomedical Research Laboratory at Aalborg University Hospital, Aalborg, Denmark. Seven landrace female pigs weighing 32.8 kg (standard deviation [SD] 2.87 kg) were used for the first series in this experiment, and 9 landrace female pigs weighing 46.47 kg (SD 8.83 kg) were used for the second series. The differences in size among the groups of pigs were not planned, but logistical conditions made us accept this situation. The reason for using only female pigs was that it is difficult to place a urinary catheter into male pigs. The animals were allowed 2 weeks of acclimation after arrival at the research facility. They were housed in groups with free access to water and feed (Altromin Spezialfutter GmbH & Co., 32 791 Lage, Germany and Special Diet Services, Essex, England).

### 2.2 Anesthesia and surgery

Anesthesia was induced with an intramuscular injection of Zoletil Vet at 2 ml/10 kg. The animals were then intubated (Portex tube 6.5 Smiths Medical, UK) and mechanically ventilated (Dameca DREAM, Roedovre, Denmark) with a tidal volume of 8 ml/kg, a fraction of inspired oxygen (FiO_2_) of 0.6, an inspiratory: expiratory ratio of 1:2, and a positive end‐expiratory pressure of 5 cm H_2_O. The respiratory rate was adjusted to maintain the partial pressure of arterial carbon dioxide (PaCO_2_) at approximately 4.5–5.8 kPa. Anesthesia was maintained with continuous infusion of propofol (4 mg/kg/h) and fentanyl (5 μg/kg/h). The infusion rate was adjusted to obtain a sufficient depth of anesthesia. Natrium chloride was infused at a rate of 10 ml/kg/h. Moreover, a bladder catheter with a thermosensor (Degania Silicone Ltd. Degania Bet 15 130, Israel) was inserted. The temperature was maintained at approximately 38°C with a forced‐air warming blanket (Mistral Air plus, The Surgical Company International B.V., Amersfoort, The Netherlands). Additionally, electrocardiogram and peripheral oxygen saturation (SpO_2_) monitoring was established.

A 6 Fr sheath (Avanti, Cordis Cashel, Ireland) was inserted percutaneously into the femoral artery for blood pressure measurements and arterial blood gas sampling (ABL 90, Radiometer Medical APS, Bronshoej, Denmark). Another 6 Fr sheath was inserted into the femoral vein on the same side as the arterial sheath. This sheath was used for medicine and saline infusion. A larger sheath (12 Fr Ultimum EV, St. Jude Medical, Plymouth, USA) was inserted into the opposite femoral vein and used for blood withdrawal to induce hemorrhagic shock, as well as for the transfusion of blood.

A 10 Fr sheath (Avanti, Cordis Cashel, Ireland) was inserted percutaneously into the right jugular vein for insertion of a pulmonary thermodilution catheter, which allowed for continuous measurement of CO and mixed venous oxygen saturation (SvO_2_) (Swan‐Ganz CCOmbo, Edwards Lifesciences Services GmbH, Unterschleissheim, Germany). The catheter was connected to an Edward Lifesciences Vigilance Monitor, model VGS2 (Edwards Lifesciences Services GmbH, Germany). The monitor was operated in the “stat” mode, in which the measurements were updated every 54 s. After insertion and calibration of the SG catheter, continuous measurements of CO were initiated. After each intervention during the experiments, the SG catheter was recalibrated by using mixed‐venous blood gases.

After sternotomy, a 16 mm transit‐time vascular probe (Medi‐Stim, Vedbaek, Denmark) was placed around the pulmonary artery. The probe was connected to a flow monitor (Medi‐Stim, Copenhagen, Denmark), thus allowing for measurements of cardiac output.

After 30 min of steady state, baseline measurements for cardiac output were conducted with both the FP and SG catheters. A total of 2500 IE heparin was administered to prevent the coagulation of blood in the catheters. The animal characteristics before experimental changes in cardiac output are presented in Table 1.

**TABLE 1:** Animal characteristics before experimental changes in cardiac output

Parameter	Series 1	Series 2
pH	7.43 (±0.05)	7.45 (±0.06)
PaCO_2_	5.2 (±0.4) kPa	5.0 (±1.1) kPa
PaO_2_	30.0 (±7.4) kPa	29.8 (±16.7) kPa
Lactate	1.3 (±1.0) mmol/L	1.0 (±0.8) mmol/L
Temperature	37.3 (±0.9) °C	37.9 (±1.2) °C
Cardiac output with Swan‐Ganz catheter	2.9 (±0.6) L/min	4.1 (±1.2) L/min
Cardiac output with transit‐time flow probe	2.5 (±0.7) L/min	3.5 (±1.2) L/min
Mixed venous blood saturation	0.73 (±0.6)%	0.66 (±0.1)%
Body weight	32.8 (±2.8) kg	46.5 (±8.8) kg

### 2.3 Study design

The experiments consisted of the following 2 parts:

#### 2.3.1 First series

The aim of this part of the experiment was to elicit a sudden doubling or halving of CO, after which the time it took to detect at least a 30% change in CO using FP and SG would be compared. First, a doubling of CO, as measured by the flow probe, was obtained via the infusion of an adrenaline solution (adrenalin, 1 mg/ml, Amgros I/S, Copenhagen, Denmark), which included 2.7 mg of adrenaline in 97.3 ml of NaCl. After 10 min or less, if both the FP and SG catheters obtained the planned level of CO, the infusion was stopped. Thereafter, baseline CO was re‐established before the next intervention. Second, a quick, controlled hemorrhage was used to decrease CO. Blood was collected in blood bags (Macopharma, Mouvaux, France) that were prefilled with citrate (CPD‐50, 250 ml, HAEMONETICS, Massachusetts, USA) to prevent coagulation. When the CO was halved, as measured by FP, the drainage was stopped. Measurements were continued every 54 s for 10 min or less if both the FP and SG catheters obtained the same change in the level of CO. After the first drainage, the blood was reinfused, and the pig baseline CO was re‐established before both interventions (doubling and halving of CO) were repeated. In this manner, more measurements could be made per animal, thus reducing the total number of animals that were used. Changes in SvO2 were registered at each measurement. After the interventions, the animals were euthanized with an intravenous injection of pentobarbiturate (Euthasol, 400 mg/ml, Virbac Danmark A/S, Kolding, Denmark).

#### 2.3.2 Second series

The aim of this part of the experiment involved more prolonged changes in CO over a period of time, in which the values of CO and SvO_2_ measured by SG and FP were compared every 15 min to determine if long‐term measurements were more consistent for the 2 methods than for those with rapid changes.

The first intervention included the infusion of the same adrenaline solution that was used in the first series (adrenalin, 1 mg/ml, Amgros I/S, Copenhagen, Denmark) until the pig had doubled its CO, as measured by the FP. Measurements were made for 30 min, after which the adrenaline infusion was stopped; thereafter, a baseline CO was re‐established.

After obtaining a steady state, CO was reduced by 50% via controlled hemorrhage. Blood was collected as in the first series. When the CO was halved, measurements were made for 30 min. After 30 min, the blood was infused again. In this manner, the pigs could be used for more than 2 interventions.

At the end of the study, the animal was euthanized with an intravenous injection of pentobarbiturate (Euthasol, 400 mg/ml, Virbac Denmark A/S, Kolding, Denmark).

### 2.4 Statistics

For the sample size calculation, the KISS approach was used,^26^ wherein the power analysis showed that a sample size of 7–9 animals would have 80% power to detect an effect size of 2–3 min, given a 5% significance level and a 2‐sided test. With the KISS approach, past experiences of sample sizes, in conjunction with table and simple calculations, are used to estimate the effect size, and the experiment is likely to detect a given power. The statistical analysis included only data from simultaneous, valid measures. All the interventions were performed in the same order by first increasing and then decreasing CO, because the risk of losing the animal was greater during the hemorrhage portion of the experiment. Thus, randomization and blinding were not possible. Exclusion criteria included inadequate calibration measurements and measurements wherein one of the methods failed.

The results were expressed either as mean ± standard deviation (SD) or median, minimum (min), and maximum (max). Owing to the considerable variability in the animals' baseline CO values, all of the changes in CO are given in percentages, thus making comparisons of these pigs possible and minimizing the number of animals that were needed for this study. The relationship between CO measured with SG catheters and CO measured with ultrasonic FP was analyzed by using linear regression, and the agreement was measured with a Bland–Altman test,^27^ but this was done only for series 2 with the prolonged tests. Additionally, a 4‐quadrant plot was drawn to demonstrate the concordance between the SG catheter and the transit‐time FP.^28^ For series 1 involving sudden changes in CO, it was obvious that the agreement between our gold standard, the transit‐time FP, and the SG catheter was poor. In all the interventions, the FP was the first to react. We presented curves with the time delay until the SG measured sudden changes in CO. Regarding bleeding, we intended to elicit only a 50% decrease in CO, as measured with FP. The curve shows the time delay for the SG to react to the intervention; we demonstrate a 30% decrease in CO during the lag of SG (as measured with FP).^21^ Given that SvO2 is only an indirect measure for CO, we described only the tendency under increases and decreases in CO. SvO2 cannot directly define CO, but we have observed that, in clinical situations, a change of 10% is considered interesting. A comparison of the rate at which the SG catheter could detect at least a 30% change in CO was performed with the Wilcoxon signed‐rank test.

Statistical analysis was performed by using the open‐source freeware program R, version 3.6.1/R‐studio and IBM SPSS, version 26. Statistical significance was set at *P* < .05. The Shapiro–Wilk analysis was used as a test for normality.

## 3 RESULTS

After sternotomy, all the animals were hemodynamically stable (Table 1). Changes in SvO_2_ often precede the CO changes that are measured with the SG catheter. As SvO_2_ is often used in the clinic and is presented together with other SG catheter measurements, the values are also presented here.

### 3.1 Series 1

One animal had ventricular fibrillation during placement of the SG catheter and was excluded from the study.

In another animal, the SG catheter could not be correctly placed with a closed thorax. After a sternotomy, the SG catheter could be guided through the heart and into the pulmonary artery.

In total, 116 paired measurements were collected from 6 pigs: 53 paired measurements during the increase in CO and 63 paired measurements during the decrease in CO. CO varied across an almost 4‐fold range (1.8 L per minute to 7.0 L per minute). Severe changes in CO lasted 6–10 min.

It was obvious that there was no clear relationship between FP and SG regarding the very abrupt changes in CO, either at the increase of CO or at the sharp decrease in CO. Moreover, the FP was able to measure changes in CO at the fastest rate.

**FIGURE 1:**
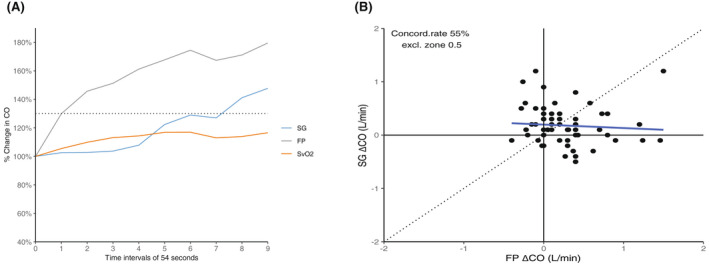
(A) The increase in cardiac output (CO) over short time changes with the measured values for the flow probe (FP), the Swan‐Ganz catheter (SG), and the mixed venous oxygenation (SvO2). (B) Four‐quadrant plot demonstrating the poor concordance rate of only 55% between the values measured with the Swan‐Ganz catheter (SG) and the flow probe (FP) during increases in cardiac output (CO).

During the increase in CO, there was a statistically significant difference in the median time it took the SG to register the 30% change, with the SG registering the time at 5.4 min (4.5–8.1 min) versus the FP at 0.9 min (0.9–3.6 min) (*P* < .05). The median time for a 10% increase in SvO_2_ was 4.5 min (0.9–9.0 min). Figure 1A demonstrates the changes in CO and SvO_2_ (in percentages) over a time period of 8 min. Measurements were conducted every 54 s. Figure 1B demonstrates the changes in a 4‐quadrant plot, wherein the concordance rate was only 55%.

During the decrease in CO, there was a statistically significant difference in the median time that it took the SG to register a 30% change in CO at 8.1 min (4.5–9.0 min) versus the FP at 2.7 min (1.8–6.3 min) (*P* < .05). The median time for a 10% decrease in SvO_2_ was 3.15 min (1.8–4.5 min).

**FIGURE 2:**
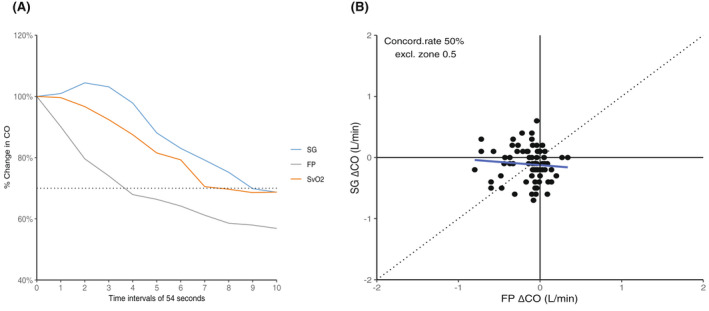
(A) The abrupt decrease in cardiac output (CO) over a short period of time with the measured values for the flow probe (FP), the Swan‐Ganz catheter (SG), and the mixed venous oxygenation (SvO2). (B) Four quadrant plot demonstrating the poor concordance rate of only 50% between the values measured with the Swan‐Ganz catheter (SG) and the flow probe (FP) during abrupt decreases in cardiac output (CO).

Figure 2A demonstrates the changes in CO and SvO_2_ (in percentages) over a 9‐min period. Measurements were taken every 54 s. Figure 2B demonstrates the changes in a 4‐quadrant plot, wherein the concordance rate was only 50%.

### 3.2 Series 2

**FIGURE 3:**
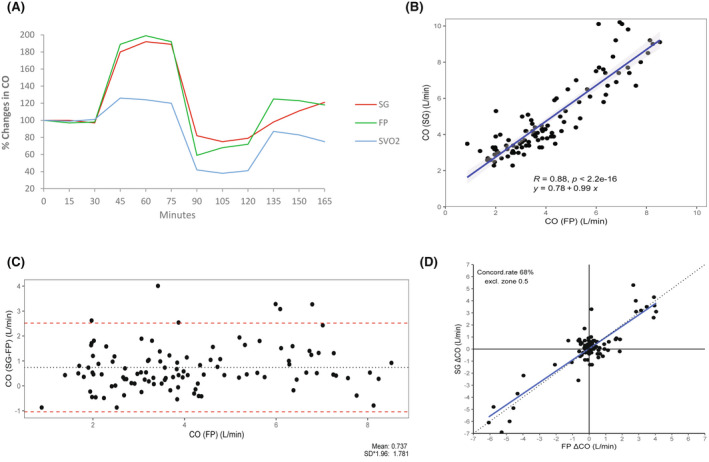
(A) Baseline cardiac output (CO) and fluctuations during long term interventions, as measured with Flow Probe (FP), Swan‐Ganz catheter (SG), and concomitant mixed venous oxygen saturation (SvO2). Each timepoint marks every 15th min measurement for each intervention. Changes are expressed in percent change from the baseline. (B) Linear regression analysis showing the correlation (R = 0.88) between the measures of cardiac output (CO) with the Flow Probe (FP) and the Swan‐Ganz catheter (SG). (C) Bland–Altman analysis of the long term tests of cardiac output (CO) measured with the Flow Probe (FP) and the Swan‐Ganz catheter (SG). The agreement showed a bias of 0.75 L/min. The SG catheter showed the highest values. (D) Four quadrant plot demonstrating the concordance rate of 68% between the values measured with the Swan‐Ganz catheter (SG) and the Flow Probe (FP) during long term changes in cardiac output (CO).

CO varied across a 5‐fold range (1.7 L per minute to 10.2 L per minute). During these lengthier experiments with changes in CO, the SG catheter better reflected the changes in CO, as the FP demonstrated. Linear regression and a Bland–Altman test showed a relatively good correlation between measurements that were made with FP compared with measurements that were made with SG. The agreement between the 2 measures showed that a bias of 0.75 L per minute was always higher for the SG than for FP, and the difference was statistically significant (*P* < .001). The 4‐quadrant plot showed a concordance rate of only 68% (Figure 3).

Of the 135 paired timepoint measurements collected from 9 pigs, the CO that was measured with the SG catheter was lower than the CO that was measured with FP in 15 of the paired measurements, which corresponded to ~10% of all the measurements. The smallest difference measured was 0.3 L, whereas the greatest difference was 3.3 L.

Each intervention lasted 30 min, with measurements made after 0, 15, and 30 min.

To increase CO, an adrenaline solution was infused; the average dose was 0.720 mg/kg (SD 0.523 mg/kg). The CO increased to 190% from baseline, according to the FP measurement.

For the reduction of CO, the animals' blood was drained. The average bleeding time was 18.33 min (SD 7.25 min). The average amount of blood that was drained was 1277.78 ml (SD 419.20 ml), and the average mean arterial blood pressure at the end of the bleeding was 39.78 mmHg (SD 6.94 mmHg). In one pig, the SG could not measure CO during the hemorrhage. CO reduction was 76% and 65% for SG and FP, respectively. After reinfusion of the drained blood, the CO increased to 118%, according to the FP.

## 4 DISCUSSION

In our human clinic, SG is the most widely used method for CO measurement. This study aimed to compare continuous cardiac output measurements from SG catheters with the golden standard, with FPs around the pulmonary artery, during acute hemodynamic instability in animal experiments. This study did not assess the speed at which other measurement methods, such as PICCO, respond to sudden changes in CO.

The difference in time for detecting changes in CO of more than 30% was 5–8 min, which consistently favored the use of the FP. This is not considered an improvement compared with the results optained from animal experiments with rapid changes in CO measured with FP and SG catheters more than 25 years ago.^4^

In many experiments, the use of FP may be too invasive, and the FP is difficult to insert and may even influence the outcome; however, in other studies, it is important to observe immediate changes in CO because the interventions may result in the death of the animal. The use of FP requires a thoracotomy, and there is a risk of arrythmia and bleeding during the insertion of the probe. In this procedure, it is possible to insert catheters for continuous pressure monitoring in the left atrium and in the pulmonary artery, thus making continuous monitoring of PVR possible.

The use of SG catheters requires minimally invasive procedures, which is why it is a widespread method for monitoring CO. It is possible to indirectly evaluate PVR with the SG catheter, but there is also a risk of bleeding, pneumothorax, and arrythmias. In many situations, it is more important to monitor changes in CO than to determine the exact CO value. The pros and cons for uses of SG catheters versus FP are listed in Table 2.

**TABLE 2:** Pros and cons for a Swan‐ganz cather or a flow probe

	Pros	Cons
Swan‐Ganz catheter	Many clinicians are accustomed to using it. Measures pulmonary blood pressure. Can indirectly indicate left atrial pressure. Measures mixed venous oxygenation. Little risk of bleeding.	Reacts slowly and not with exact values. Risk of arrhythmias.
Flow probe	Measures cardiac output beat‐to‐beat. Reacts almost immediately.	Highly invasive, requires surgical skill. Risk of bleeding.

Less invasive methods for CO monitoring have been described, such as echocardiography; however, they are dependent on the constant presence of trained personnel.^15^ In some cases, capnodynamic examinations may be beneficial, similar to extracorporeal arteriovenous ultrasound measurements, especially in smaller animals or children; however, their use is still not widespread.^18^, ^19^

We chose a detection level of a 30% decrease or increase in CO according to Critchley and Critchley.^21^ Continuous measurements of SvO_2_ have long been accepted as a reliable method to estimate global oxygen delivery and consumption. Sudden changes in SvO_2_ could be an early indication of cardiorespiratory failure.^29^ However, large changes in SvO_2_ are not commonly expected because they are measured as a percentage. Therefore, we chose to monitor the detection of a 10% increase or decrease in SvO_2_ instead of the 30% changes in CO. SvO_2_ changes are only a warning of a problem; they do not directly reflect the CO value.

In series 2, wherein the changes in CO were slower, the relationship between the SG catheters and FP was acceptable, and most of the literature describing the accuracy of SG catheters has described patients who were admitted for a longer time period in an intensive care unit. These studies did not describe experiments with sudden changes in CO.^30^

Developments in CO measurements are still ongoing; very recently, Edwards Lifesciences launched a new SG catheter that measures CO every 20 s instead of every 54 s. Furthermore, the device can be combined with pulse wave analysis. We do not know if this new catheter will reduce the difference between an SG catheter and a FP around the pulmonary artery. Thus, future experiments may be necessary to demonstrate this effect.

There were several limitations to the study. For example, this was a small‐scale animal study. In addition, the fast reduction in CO was artificial with massive bleeding, which may interfere with the SG measurements, although no simultaneous infusion of cold fluid was performed. Finally, there were different body weights in the 2 groups.

## 5 CONCLUSION

In these animal experiments with sudden changes in CO that were measured with the SG catheter, the reactions were delayed by 5–8 min. FP reacted immediately, but its use requires invasive surgery. For more prolonged experiments, the relationship between the 2 methods was better.

## CONFLICT OF INTEREST

The authors declare that they have no competing interests.

## AUTHORS’ CONTRIBUTIONS

B.K. and B.S.R. developed the main research idea and supervised the project. S.O.M., C.S., B.S.R., and B.K. performed the surgeries together and collected all the data. P.E.L. and S.O.M. processed and performed the data analysis. S.O.M. and B.K. interpreted the results. S.O.M. wrote the first draft of the paper. All the authors assisted in drafting the manuscript and approved the final document.

## ETHICS APPROVAL

This study was performed in accordance with Danish and European legislation regarding the use of animals for research purposes. The Danish National Animal Ethics Committee approved the experiments (license no. 2016‐15‐0201‐00930).

## Data Availability

The datasets used and/or analyzed during the current study are available from the corresponding author on reasonable request.
